# Antiviral Activity of a Zymolytic Grain Based Extract on Human Immunodeficiency Virus Type 1 *In Vitro*

**DOI:** 10.1155/2015/642327

**Published:** 2015-03-16

**Authors:** Chu Wang, Di Liu, Xiao-Han Guo, Bin Yu, Hui Wu, Hai-Hong Zhang, Jia-Xin Wu, Chun-Lai Jiang, Wei Kong, Xiang-Hui Yu

**Affiliations:** ^1^National Engineering Laboratory for AIDS Vaccine, School of Life Sciences, Jilin University, Changchun 130012, China; ^2^Key Laboratory for Molecular Enzymology and Engineering, the Ministry of Education, School of Life Sciences, Jilin University, Changchun 130012, China

## Abstract

Increasing evidence shows that grains may play a role in disease prevention beyond the simple provision of energy and nutrients. It has been reported that some components contained in grains exert their functional effects on viral and bacterial infections and protect against various cancers. However, until now, hardly any intervention studies have investigated the effects of grains or grain based extracts on the inhibition of HIV-1 infection. In this study, the antiviral function of a zymolytic grain based extract (ZGE) was detected *in vitro* and in rats, and the antiviral mechanism was investigated. Results showed that ZGE had an inhibition effect on HIV-1 infection *in vitro* with low cytotoxic effects. The study of the mechanism demonstrated that this functional food possibly acted on the viral surface structure protein gp120 which is responsible for cell binding, as well as on the postattachment stage of the virus. The sera of model rats administrated with this food by gavage presented anti-infection abilities against HIV-1 *in vitro* during a serum concentration associated period of time. These findings provide valuable insights into the application of ZGE on the control of viral load, which may contribute to future anti-HIV treatment with less adverse effects.

## 1. Introduction

Grain is indispensable to human since ancient times, not only providing nutrients as food but also benefiting health. Based on previous studies, greater whole-grain intake is associated with lower risk of some diseases such as type 2 diabetes (T2D), cardiovascular disease (CVD), and weight gain [1–3]. Intake of diverse grains containing large amounts of fiber in dietary is known to exert various beneficial effects on human health. Many kinds of grains, like black rice, purple rice, and rice bran, were intensely studied for their anticancer [4], antioxidative [5, 6], anti-inflammatory [7], and antidiabetic effects [8, 9]. However, very few studies have been carried out to divulge the effects of grains against viral infection, especially the infection of the human immunodeficiency virus type 1 (HIV-1).

HIV-1 is the pathogen of the acquired immunodeficiency syndrome (AIDS) in humans, with the depletion of CD4^+^ lymphocytes, the major target cells of viral infection* in vivo*, eventually resulting in defective cellular immunity [10]. As a retrovirus, HIV-1 delivers its RNA genome into cytoplasm after membrane fusion of the virus and target cells. This entry step is mediated by the surface glycoprotein gp120 and the transmembrane glycoprotein gp41 which are yielded by cleavage of a HIV envelope (Env) protein (gp160) [11]. Cellular coreceptors CCR5 or CXCR4 are engaged in the binding process between gp120 and cell surface. A subsequent conformational change of gp41 impels viral core to enter the target cell [12, 13]. The RNA genome is then converted to cDNA form through the process of reverse transcription (RT), followed by the integration of cDNA into the host cell DNA genome. The complex, multistep HIV replication cycle offers numerous opportunities for pharmacological intervention and highlights the urgent need for effective antiviral agents to reduce the rate of drug resistance and suppress viral replication [14]. A system has been established to evaluate the anti-HIV-1 activity of drugs and its kinetics in low-cost animal models [15]. The inhibition effect of animal sera on HIV infection was investigated by viral infectivity assay based on HIV infection indicating cells.

In order to take advantage of the beneficial effects of different grains, a mixture of nine ingredients was processed by zymolysis with a compound of enzymes to produce a zymolytic grain based extract (ZGE). Previous research found that ZGE could reduce gastrointestinal infections and assist with the treatment of dyspepsia and patients with impaired immunity. The present study was therefore undertaken to investigate the suppressive efficacy of ZGE on HIV-1 by performing viral infectivity assay* in vitro* and gavage administration on rats. The possible mechanism of the antiviral activity of ZGE was also demonstrated. The identification of ZGE as a nutraceutical initially reveals its potential for adjuvant treatment for AIDS with less adverse effect.

## 2. Methods and Materials

### 2.1. ZGE Samples

ZGE products were provided by the Tianjin Quanyoujin Biological Technology co., LTD., in China. A standard manufacturing process was strictly followed and the conditions of stabilization were submitted as patent (number ZL99107906.X). ZGE was produced by using white rice (15%, w/w), black rice (10%, w/w), purple rice (10%, w/w), sticky rice (15%, w/w), fragrant rice (10%, w/w), sorghum rice (10%, w/w),* Coix* seed (10%, w/w), millet (10%, w/w), and broomcorn millet (10%, w/w) as raw materials. The steamed and gelatinized grains were mixed with a compound of glucoamylase (0.25%), lipase (0.05%), protease (0.05%), and amylase (0.02%) for saccharification and zymolysis at 25–35°C. This process ended when the content of total sugar (by reducing sugar) and total acid (by succinic acid) was reduced to 16–22 g/mL and 0.38–0.6 g/mL. The zymolytic grains were then crushed, separated to remove dregs, and finally made into a homogeneous and sterile fluid food. The nutrition facts of ZGE are shown in Table 1.

### 2.2. Cells, Plasmid, and Reagents

HEK293T (CRL-11268) cells were purchased from the American Tissue Culture Collection (ATCC). MAGI-CCR5 cells (catalog number 3522) and TZM-bl cells (catalog number 8129) were obtained from the National Institutes of Health AIDS Research and Reference Reagents Program (NIH-ARRRP), Division of AIDS, National Institute of Allergy and Infectious Diseases (NIAID). HEK293T, MAGI-CCR5, and TZM-bl cells were cultured in Dulbecco's modified Eagle's medium (DMEM) supplemented with 10% fetal bovine serum (FBS) at 37°C in 5% CO_2_. The infectious molecular clone pNL4-3, anti-HIV-1 Env antibody 3B3, HIV-1 gp41 monoclonal antibody 4E10, and nucleoside reverse transcriptase inhibitor lamivudine (3TC) were obtained from NIH-ARRRP. Pr55Gag and CAp24 were detected with a monoclonal anti-HIV capsid antibody generated by an HIV-1 p24 hybridoma (NIH-ARRRP). Anti-tubulin antibody was purchased from Covance.

### 2.3. Transfection and Virus Purification

Plasmid transfections into HEK293T cells were carried out using Lipofectamine 2000 (Invitrogen) as recommended by the manufacturer. Viruses in cell culture supernatants were harvested 48 h after transfection and precleared of cellular debris by centrifugation at 3,000 rpm for 10 min and filtration through a 0.22 *μ*m-pore-size membrane. Virus particles were then concentrated through a 20% sucrose cushion by ultracentrifugation at 100,000 g for 2 h at 4°C and resuspended in sterilized PBS.

### 2.4. Western Blotting Analysis

Cells were collected 48 h after transfection and lysed with RIPA buffer. Cell lysates or purified virions were mixed with 4X sample loading buffer and boiled for 10 min, subjected to standard SDS-PAGE, and transferred to nitrocellulose membranes. The mouse monoclonal antibodies (MAbs) were used at a 1/1000 dilution and the ImmunoPure alkaline phosphatase-conjugated goat anti-mouse IgG secondary Abs (Jackson ImmunoResearch Laboratories, Inc.) were used at a 1/10,000 dilution. The color reaction was performed with 0.1 M Tris-HCl (pH 9.5) containing 0.66% nitro blue tetrazolium (NBT) solution and 0.33% 5-bromo-4-chloro-3-indolyl phosphate (BCIP) solution.

### 2.5. Viral Infectivity Assay

Viral infection was determined by a multinuclear activation of either a galactosidase indicator (MAGI) assay or a luciferase indicator (TZM-bl) assay as previously described [16, 17]. In general, MAGI-CCR5 or TZM-bl cells were plated at 100,000 per well in 24-well plates or at 10,000 per well in 96-well plates one day before infection. Virus input was normalized by the level of reverse transcriptase (RTase) (Lenti RT Activiy Kit, CAVIDI). Viruses with equal RTase units were mixed with ZGE samples and then incubated with MAGI-CCR5 or TZM-bl cells for 1.5 h at 37°C, followed by addition of complete DMEM (DMEM with 10% FBS). After incubation for 48 h, supernatants were removed. For MAGI-CCR5 cells, 5-bromo-4-chloro-3-indolyl-*β*-D-galactopyranoside (X-Gal) was added as the substrate for *β*-galactosidase. Positive blue dots were counted to determine viral infectivity. For TZM-bl cells, the substrate for luciferase was added and the expression of luciferase was quantified using the Steady-Glo Luciferase Reagent System to determine viral infectivity. Each experiment was performed in triplicate.

### 2.6. Cytotoxicity Assay

MTT [3-(4,5-dimethylthiazol-2-yl)-2,5-diphenyltetrazolium bromide] was used to assess cytotoxicity of ZGE samples. Exponentially growing MAGI-CCR5 or TZM-bl cells were plated at 1,000 or 2,000 per well in 96-well plates and cultured for 24 h. A 200 *μ*L aliquot of sample solution, diluted with complete DMEM, was added to the cells and incubated for 44 h. After the supernatants were removed, MTT solution was added to each well and incubated for 4 h. The absorbance at 490 nm was measured using a multiwell plate reader. The DMEM-treated cell group was set as 100%. Each experiment was performed in quintuplicate.

### 2.7. Drug Blocking Stage Analysis

TZM-bl cells were plated at 100,000 per well in 24-well plates and cultured overnight. Before infection, the cells were pretreated at 4°C in the presence or absence of ZGE samples (6.25%, *μ*L/*μ*L), 3TC (100 *μ*M) or 4E10 (25 *μ*g/mL) for 1.5 h, and then washed three times with DMEM. Next, the cells were incubated with RTase-normalized viruses alone or with preincubated or immediately mixed solutions containing the viruses and one of the three drugs above at 4°C for 1.5 h. After being washed another three times, the cells were cultured with complete DMEM alone or containing the corresponding drug at 37°C for 48 h (as shown in Figure 3(a)). Finally, the harvested cells were performed with the luciferase indicator assay to determine viral infectivity as mentioned in Section 2.5.

### 2.8. Experimentation on Animals

Two groups of six male SD rats (7 weeks, 215.9 ± 8.2 g) were used to examine the pharmacological kinetics of ZGE in sera. The experiment was carried out under the “Guidelines and Regulations for the Care and Use of Laboratory Animal” of the Science and Technology Commission of Jilin Province, China. ZGE samples (10 mL/kg) and the same volume of normal saline (NS) were administrated to the two groups of rats by gavage. Sera were then harvested at 0.5, 1, 2, 4, and 8 h after administration and used within 1-2 days. 1/20-diluted sera were added to TZM-bl cells in triplicate with HIV-1 preparations. After 48 h, cultured cells were harvested and performed with the luciferase indicator assay as mentioned in Section 2.5.

### 2.9. Statistical Analysis

Values are presented as means ± SD. The statistical significance between groups was examined by Student's* t*-test or repeated-measures analysis of variance (ANOVA) followed by the post hoc Tukey's test (^*^ *P* < 0.01, ^**^ *P* < 0.005, and ^***^ *P* < 0.001).

## 3. Results

### 3.1. Anti-HIV-1 Activity of ZGE* In Vitro*

**Figure 1:**
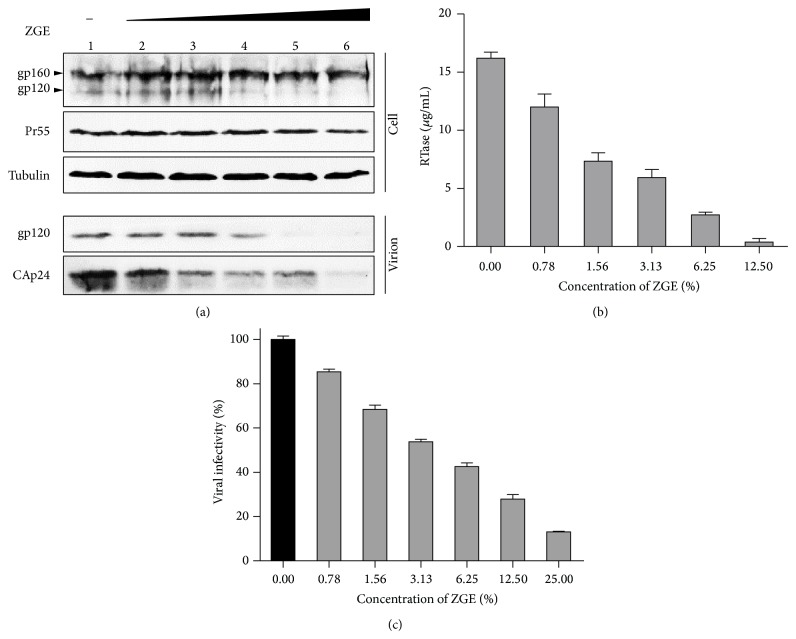
Effect of ZGE on HIV-1 infectivity. (a) ZGE suppressed the output level of the virus with increased concentrations (lanes 1–6, 0.00, 0.78, 1.56, 3.13, 6.25, and 12.50%). (b) Viral output was quantified by the level of RTase at each concentration of ZGE. (c) ZGE showed dose-dependent inhibition of viral infectivity in MAGI-CCR5 cells, with viral infectivity in control DMEM set to 100% (black column). Error bars represent the standard deviation (SD) calculated from three independent infections.

HEK293T cells were transfected with a pNL4-3 vector and then cultured with control DMEM or serially diluted ZGE-DMEM solution from 0.00 to 12.50% (*μ*L/*μ*L). Protein levels in cell lysates and purified virions were analyzed by Western blotting at 48 h after transfection (Figure 1(a)). It could be observed that the expression level of viral proteins in cell lysates was not interfered by ZGE, while the output level of virions was significantly suppressed. The direct quantification of viral output by the level of RTase of HIV-1 was shown in Figure 1(b). To investigate the effect of ZGE on the infection of HIV-1, viruses with equal RTase units were used to infect MAGI-CCR5 cells. Viral infectivity was tested in a standard MAGI assay as previously described [18]. The relative infectivity of virus mixed with serially diluted ZGE was calculated based on the infectivity of DMEM-treated virus set to 100% (Figure 1(c)). ZGE specifically reduced the infectivity of viruses produced from HEK293T cells in a dose-dependent manner. From these data, ZGE demonstrates a strong inhibitory effect on HIV-1 as we expected.

### 3.2. Analysis of the Components, pH, and Cytotoxicity of ZGE

**Figure 2:**
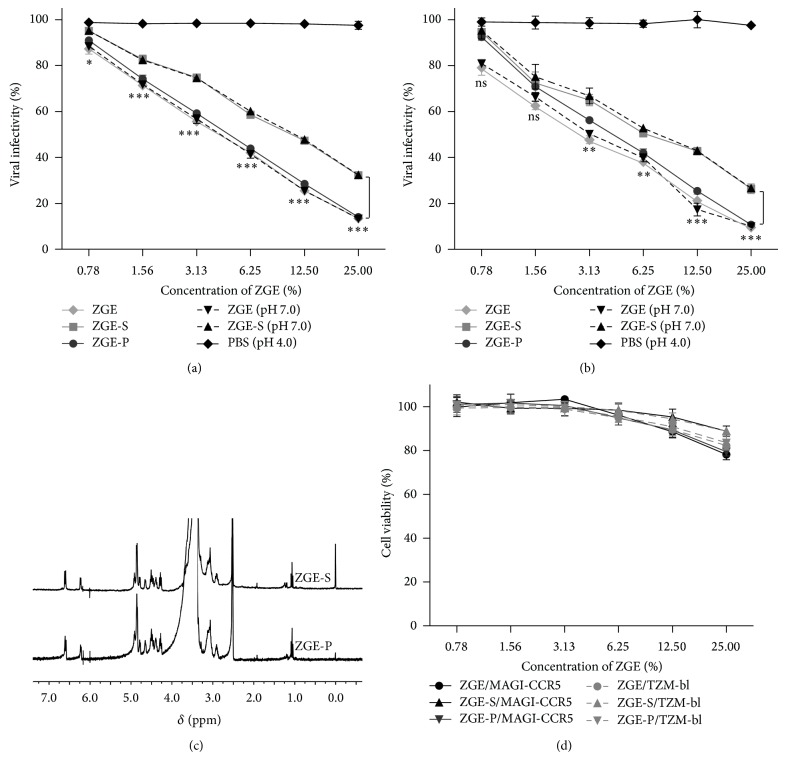
Anti-HIV-1 activity of ZGE comes from grain component(s) and is independent on its acidic property. (a) MAGI-CCR5 cells were infected with the prepared virions mixed with the indicated concentration of ZGE, ZGE-S, ZGE-P, ZGE (pH7.0), ZGE-S (pH7.0), PBS (pH4.0), or control DMEM. Viral infectivity was tested in a MAGI assay. (b) A repeated assay on TZM-bl cells as (a). Viral infectivity was tested in a luciferase indicator assay. Results in (a) and (b) are the average of three independent experiments. The statistical significance was tested between the ZGE-S group and ZGE-P group. (c) ^1^H NMR spectra of ZGE-S and ZGE-P. Chemical shifts (*δ* in ppm) were determined with a residual proton of the solvent (DMSO) as standard. (d) Cell viability was assessed after 48 h treatment with different concentrations of ZGE, ZGE-S, and ZGE-P by using an MTT assay.

As ZGE was a kind of fluid food containing insoluble materials from grain, we wondered whether there was a difference between the liquid and solid components for its anti-HIV-1 activity. Additionally, given that the natural pH of ZGE was 4.0 which might influence the infectivity of HIV-1 as several previous reports had described the acid sensitivity of this virus [19, 20], we prepared neutral ZGE samples (NaOH adjusted) and carried out the viral infectivity assay in both MAGI-CCR5 cells and TZM-bl cells (Figures 2(a) and 2(b)). The supernatant (ZGE-S) and precipitation (ZGE-P) of ZGE were separated by centrifugation at 12,000 g for 5 min. The precipitation was resuspended with the same volume of distilled water as the separated supernatant. It could be observed that ZGE-S demonstrated lower anti-HIV-1 activity compared with ZGE-P in both cells. However, low pH was not a main factor in inhibiting viral infection as there were no significant differences between the original pH and the pH 7.0 groups of ZGE and ZGE-S. From these data we determined ZGE, ZGE-S, and ZGE-P to have a 50% inhibitory concentration (IC50) of 7.91%, 14.65%, and 8.84% (*μ*L/*μ*L) by using MAGI-CCR5 cells and 5.36%, 11.95%, and 8.07% (*μ*L/*μ*L) by using TZM-bl cells, respectively.

To investigate the difference of anti-HIV-1 activity between the liquid and solid components, ^1^H NMR spectra were recorded on a Varian Mercury-300 NMR spectrometer for the detection and identification of ZGE-S and ZGE-P (Figure 2(c)). The spectra showed no distinct difference between them, indicating that the lower activity of ZGE-S may result from the lower concentration of active component(s) it contains compared to the solid component. Both MAGI-CCR5 and TZM-bl cells used in our experiments were tested with ZGE, ZGE-S, and ZGE-P for cytotoxicity at their active concentrations (Figure 2(d)). Result demonstrated that these samples showed little adverse effects on cell viability. MAGI-CCR5 and TZM-bl cells treated with 0.78 to 6.25% ZGE samples for 48 h mostly had viabilities greater than 95%. Even in the highest concentration group treated with 25.00% ZGE, viable cell rates remained nearly 80%. Based on a comprehensive consideration of anti-HIV-1 activity and cytotoxicity, the concentration of ZGE at 6.25% was selected to perform the following experiment to investigate its mechanism in anti-HIV-1 activity.

### 3.3. Mechanism of the Anti-HIV-1 Activity of ZGE

**Figure 3:**
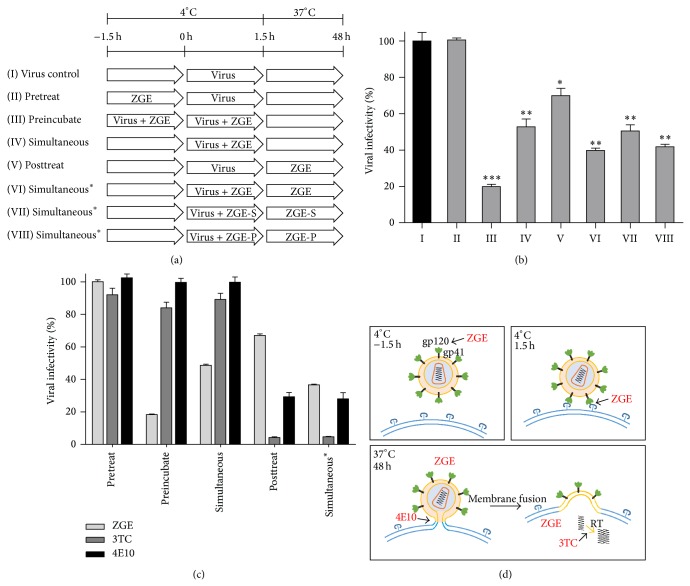
ZGE acts on both the structural protein on viral surface and the post-attachment stage of viral infection. (a) A diagram of the drug blocking assay. During the −1.5 h, TZM-bl cells and the prepared virions were incubated with ZGE respectively at 4°C in Procedure IIand III. During the following 1.5 h for viral attachment, the viruses alone (I, II, and V) or with ZGE (IV and VI), ZGE-S (VII), or ZGE-P (VIII) were added to the cells. The already incubated viruses with ZGE in Procedure (III) were added as the others. Finally, the cells wereincubated in the absence (I–IV) or presence of ZGE (V and VI), ZGE-S (VII), or ZGE-P (VIII) until harvested. (b) Relative infectivity of viruses in the procedures shown in (a) was tested in a luciferase indicator assay, with the viruses in Procedure (I) set to 100% (blackcolumn). Results shown are the average of three independent infections. The statistical significance was tested between the virus control group and other groups. (c) ZGE, 3TC and 4E10 were tested for their blocking stages according to the procedures described in (a). (d) Model of drug blocking at different stages in the process of HIV-1 attachment and membrane fusion. (Upper left) ZGE may act on the HIV-1 Env surface subunit gp120 via the 1.5 h incubation with the viruses, resulting in the inhibition of viral infectivity. (Upper right) gp120 binds with the coreceptors on the target cell at 4°C. ZGE continues to interfere with this process. (Lower) The HIV-1 Env transmembrane subunit gp41 does not change conformation to expose the epitope that 4E10 binds to until the temperature is shifted to 37°C. Thus 4E10 functions at the post-attachment stage. The HIV-1 envelope fuses withthe cytoplasmic membrane and viral RNA is released into the cytoplasm where 3TC functions to inhibit the process of reverse transcription (RT). ZGE also has effects on the post-attachment stage where the mechanism is not clear yet.

The process of HIV-1 entry can be subdivided into several stages according to the status of viral attachment. A modified drug blocking stage analysis [21] was carried out to determine the stages of HIV-1 replication cycle targeted by ZGE. As shown in Figure 3(a), the assays were performed according to eight different procedures: (I) as control, the cells were first exposed to HIV-1 at 4°C for 1.5 h to allow virus attachment to cells. The unattached viruses were then removed and complete DMEM was added for culturing the cells at 37°C until harvest. The infectivity of these nontreated viruses was set to 100%; (II) the cells were incubated with ZGE before viral exposure. A positive effect suggests it is targeting the preattachment stage; (III) the viruses were preincubated with ZGE at 4°C for 1.5 h and then the mixture was added to the cells for another 1.5 h to allow viral attachment. A positive result in this procedure suggests that ZGE could directly inactivate the viruses; (IV) the viruses and ZGE were added to the cells simultaneously without the preincubation described in (III); (V) after the viruses had attached to the cells and were removed by washing, ZGE was added and incubated with the cells until harvest. A positive effect indicates that ZGE is most likely targeting the postattachment steps of viral exposure; Procedures (VI), (VII), and (VIII) involved a simultaneous effect combining (IV) with (V) and tested the activities of ZGE, ZGE-S, and ZGE-P, respectively. Positive results could be observed in Procedures (III) to (VIII) (Figure 3(b)). The strongest blocking effect (viral infectivity was reduced to 19.84 ± 1.36%) was detected when the viruses had been preincubated with ZGE as in Procedure (III). The weaker effect presented in (IV) mostly resulted from the lack of preincubation, and the enhanced result observed in (VI) was considered as a combined effect from the two-staged addition of ZGE. The activities of ZGE-S and ZGE-P were consistent with that obtained in Figure 2(b). These data suggest that ZGE inhibits HIV-1 by both inactivating the virus and targeting the postattachment stage of viral infection.

To further understand the mechanism of ZGE against HIV-1, cytosine analogs lamivudine (3TC) targeting viral reverse transcriptase [22] and broadly neutralizing antibody 4E10 targeting gp41 [23] were assayed according to Procedures (II) to (VI) described in Figure 3(a) (Figure 3(c)). The viral infectivity was dramatically decreased by the inhibition of 3TC in the postattachment stage, and the nonignorable effect of 3TC via incubation of the cells in presence or absence of the viruses (corresponding to Procedures (II) to (IV)) might result from a spontaneous drug diffusion into the cells without virus-associated processes. On the other hand, 4E10 binds to gp41 upon its epitope exposure which may occur after the initial attachment accomplished by gp120, thus inhibiting gp41-mediated membrane fusion. Result showed that incubation with the viruses at 4°C by 4E10 did not affect the viral infectivity, therefore indicating the conformation changes of gp41 did not initiate until the temperature was risen to 37°C. Compared to 4E10, it can be deduced that ZGE mainly acts on the process of HIV-1 binding to cell surface receptors prior to membrane fusion, and the target may be the viral surface glycoprotein gp120. The staged impacts of ZGE on HIV-1 infection are illustrated in Figure 3(d).

### 3.4. Anti-HIV-1 Activity in Rats

**Figure 4:**
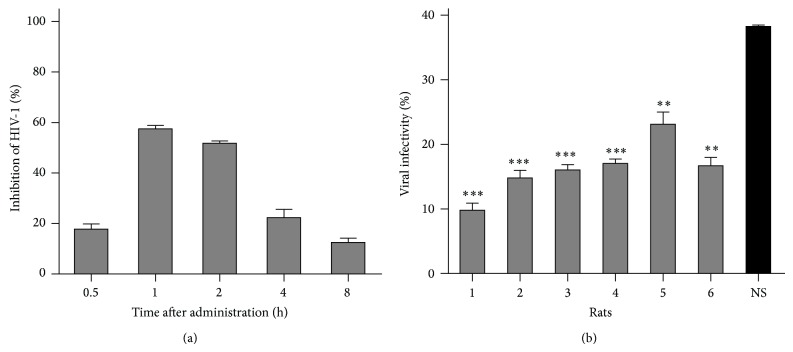
Anti-HIV-1 activity of ZGE in rats. (a) Two groups of rats were administered with ZGE or normal saline (NS) by gavage. Rat sera were harvested at different time points postadministration and analyzed by the luciferase indicator assay. Data represent mean ± SD from six rats. This experiment was performed in triplicate for each rat. Relative inhibition rates of the viral infectivity were calculated at each time point between the ZGE group and NS group. (b) The anti-HIV-1 activity from the serum of each ZGE-administrated rat was compared with the mean activity of the NS group rats (black column) at 1 h postadministration. The statistical significance was tested between the data of NS group and that of each rat.

The anti-HIV-1 activity of ZGE was further confirmed by an evaluation system based on rat model. ZGE and NS were administrated by gavage to two groups of rats (*n* = 6) and sera were withdrawn at the indicated time as shown in Figure 4(a). The sera were diluted at 1/20 and added to TZM-bl cells in triplicate with HIV-1 preparations. A relative inhibition rate of HIV-1 was shown by calculating the ratio of the viral infectivity of ZGE group to that of NS group at each time point, with a peak time point 1 h after administration. The inhibition activity at the peak time point nearly reached up to 60%. Additionally, the anti-HIV-1 activity in the serum of each ZGE-administrated rat at 1 h postadministration was shown in Figure 4(b) and a stable effect could be observed. These results indicate that ZGE can inhibit HIV-1 infection* in vivo* even after gastrointestinal digestion and absorption.

## 4. Discussion

There is an increased amount of evidence showing that consumption of whole grains and whole-grain-based products is associated with a reduction of the risk of developing many diseases, including T2D, CVD, hypertension, and different types of cancer [1–3, 24–26]. The mechanisms by which whole grains exert their benefits are not fully understood, but the main chemical components, such as fiber, *β*-glucan, and vitamin E, which may exert functional effects have been discussed [27]. Anthocyanins, flavonoids, and phenolic acids are likely candidates for the anticancer, antimicrobial, and antioxidant activity in cereal and plant extracts [28, 29]. In our study, ZGE is a kind of grain based product containing nine different grains treated by zymolysis with a compound of enzymes. The effect of it could not be distinguished between the liquid phase and the solid phase as shown in Figure 2(c). Therefore, mass spectrometry (MS) and high performance liquid chromatography (HPLC) can be utilized to further identify and isolate each component. According to our preliminary results obtained by HPLC, polysaccharides, oligopeptides, and other candidates at considerably high concentrations in the chromatogram were selected for further analysis. However, it is necessary to be aware that the anti-HIV-1 activity of ZGE may not only rely on a certain component. Synergistic effects and interactions between these components may be more important.

Another concern about ZGE is its blocking stage against HIV-1. The general steps of HIV-1 entry are mediated by a viral fusion protein Env which is posttranslationally cleaved into gp120 that mediates receptor binding and that remains noncovalently attached to gp41 [11, 30]. gp120 triggers the conformation changes of gp41 to accomplish fusion of the viral membrane with the target cell membrane. The broadly neutralizing antibodies targeting the membrane proximal external region (MPER) of gp41, such as 2F5 and 4E10, can prevent refolding of gp41 into the six-helical bundle conformation, thus inhibiting gp41-mediated membrane fusion [23, 31, 32]. MAbs 2F5 and 4E10 require some level of conformational rearrangement of MPER to release critical residues within the core epitope. Unlike 2F5, the epitope peptide that 4E10 binds to is immersed in the interfacial region of the lipid bilayer [33]. Kinetic data showed a temperature-arrested state in fusion related events in which 4°C incubation did not disturb the engagement of coreceptors by virion-associated gp120, whereas binding sites within gp41 were not exposed until after temperature had been increased [34]. This is consistent with our result by using 4E10 as gp41 inhibitor and further provides an evidence that the component(s) in ZGE might interact with the structural protein on viral surface, which was inferred to be gp120, leading to the inactivation of viral particles. On the other hand, we also showed the function of ZGE in postattachment stage. This effect may come from other component(s) in it and is urgent to be explored by addition of ZGE at various time intervals after attachment.

ZGE also showed significant anti-HIV-1 effects in the animal model in the present study. However, sera themselves caused a relatively high background that only 39% of viruses could infect cells in the group of rats administrated with NS (Figure 4(b)). This background might be caused by nonspecific inhibitory component(s) in sera. For instance, serum albumin, lactoferrin, and transferrin may influence HIV replication as previously reported [35–38]. To improve the accuracy and sensitivity of this evaluation model of* in vivo* ZGE efficacy, administration of relatively high doses of it and further dilution of sera may be required.

Although it was reported that certain foods, such as oat bran, soy protein, and flaxseed, were shown to help patients with HIV/AIDS control metabolic disorders resulting from antiretroviral treatment [39], to our knowledge, none of foods could directly act on the viral particles, followed by reducing the viral infectivity. The application of ZGE products provides valuable insights into the treatment of patients with AIDS. The increasingly complex drug therapy for HIV-infected patients is able to be cut down by combining this food with low cytotoxic effects.

## 5. Conclusion

In this study, we identified a nutraceutical, ZGE, which had an inhibition effect on HIV-1 infection* in vitro* and* in vivo* with low cytotoxic effects. The anti-HIV-1 activity of this food was independent of the acidic property of it. The possible mechanism was that ZGE acted not only on the viral surface structures being responsible for cell binding, but also on the postattachment stage of the virus. Thus, ZGE is a unique food that can assist with the treatment of AIDS.

## Figures and Tables

**Table 1:** Main ingredients and nutrients of ZGE.

Carbohydrate	18.0% (by weight)	Iron	51 mg/L
Protein	4.7% (by weight)	Magnesium	240 mg/L
Amino acid	19 g/L	Zinc	23 mg/L
Nucleic acid	7.6 g/L	Cuprum	0.8 mg/L
Vitamin (B_1_ + B_2_ + B_6_)	29 *μ*g/L	Manganese	3.0 mg/L
Niacin	50 *μ*g/L	Selenium	0.014 mg/L
Calcium	320 mg/L	Potassium	250 mg/L
Phosphorus	356 mg/L	Sodium	580 mg/L
